# Public preferences for preventative dental care: a discrete choice experiment in China

**DOI:** 10.3389/fpubh.2026.1886047

**Published:** 2026-08-31

**Authors:** Dan Yang, Xiuxian Li, Bining Chen, Bixia Liu, Zhongcai Huang, Meirong Qin, Weiju Chen, Yonglan Tang, Xiaohong Wu, Jinsu Chen

**Affiliations:** 1 School and Hospital of Stomatology, Guangdong Engineering Research Center of Oral Restoration and Reconstruction & Guangzhou Key Laboratory of Basic and Applied Research of Oral Regenerative Medicine, Guangzhou Medical University, Guangzhou, China; 2 Zhongshan Third People's Hospital, Zhongshan, Guangdong, China; 3 School of Nursing, Jinan University, Guangzhou, Guangdong, China

**Keywords:** general population, preference, preventative dental care, willingness to pay, willingness-to-pay estimates

## Abstract

**Introduction:**

The traditional treatment-oriented model is no longer sufficient to address the growing global burden of oral diseases. Although preventative oral health services effectively reduce the burden of oral diseases, their utilization remains suboptimal. Understanding the public preferences regarding preventative dental care (PDC) is essential to improve service efficiency and optimize preventative care programs.

**Methods:**

A discrete choice experiment (DCE) questionnaire was distributed via face-to-face interviews to 277 participants recruited from three stomatology hospitals in South China. Six key attributes of PDC were identified. Mixed logit models were used to estimate the preferences for each attribute, while relative importance scores (RIS) and willingness-to-pay estimates (WTP) were calculated. Latent class analysis (LCA) was used to identify distinct preference patterns.

**Results:**

Hospital type, risk of adverse reaction, service effectiveness, cost, attitude of provider, and care provider all significantly influenced respondents’ preferences. Hospital type was the most influential attribute. Respondents were willing to pay an additional RMB 1581.90 to upgrade from small private clinics to class A tertiary hospitals. Latent class analysis identified three distinct preference classes.

**Conclusion:**

This study explores the PDC preferences of urban Chinese adults from demand-side perspective. Medical institutions should prioritize public preferences when redesigning PDC programs, thereby enhancing the acceptance rate of PDC.

## 1 Introduction

Oral diseases represent a major global public health burden, affecting 3.69 billion people and accounting for approximately 23.22 million disability-adjusted life years (DALYs) worldwide in 2021 (1). According to the World Economic Forum’s 2024 report *The Economic Rationale for a Global Commitment to Invest in Oral Health*, the annual costs of treatment and productivity losses attributable to oral diseases exceeded US$710 billion (2). China bears a particularly heavy burden of oral diseases. Nearly half of its population suffers from oral diseases, and Chinese oral disease-related DALYs accounted for 18.95% of the global total (reaching 4.4 million) in 2021 (3, 4). Consequently, shifting from a treatment-oriented model to a prevention-oriented model has become a global priority for improving oral health systems (5, 6).

Preventative dental care (PDC) is essential for reducing the burden of oral diseases. In this study, PDC refers to professional mechanical plaque removal and oral hygiene advice delivered by dental care facilities (7). Evidence demonstrates that regular utilization of preventative dental services (once or twice annually) improves oral health outcomes and quality of life (7–11). Routine PDC reduces gingivitis and prevents progression to periodontitis by removing plaque and calculus (tartar) from tooth surfaces (12). Beyond clinical benefits, PDC also improves psychosocial well-being by preserving dentofacial aesthetics and social confidence (13). Despite these well-documented benefits, consistent engagement with PDC remains inadequate across global populations (9, 14–16). A systematic review covering 28 countries estimated a global average preventative dental service uptake rate of 54% (50–59%) (17). This gap is far more pronounced in China. The Fourth National Oral Health Survey of China reported that only 0.7–8.5% of Chinese adults sought preventative oral care (4), revealing a substantial gap between oral health needs and utilization of dental services. Previous studies have indicated that the uptake of PDC is influenced not only by service availability and quality but also by individual-level factors, including oral health literacy, socioeconomic status, and cultural context (17–20). However, most existing studies have primarily examined determinants of service utilization while paying limited attention to public preferences regarding preventative dental services. Understanding these preferences is essential for developing patient-centered service models and designing strategies to increase the uptake of preventative dental care. A discrete choice experiment (DCE) is a stated-preference measurement method grounded in demand theory and the random utility theory (21). This method is increasingly applied in the field of health economics and policy research (22, 23). Previous DCE studies have investigated preferences for PDC across different national contexts (7, 24, 25). Boyers et al. found that the general population in the UK preferred more frequent professional mechanical plaque removal, care provided by a dentist, and personalized oral hygiene advice (7). Bahanan et al. found that adults living in Saudi Arabia identified the ability to get an appointment as the most important driver of preventative dental visit decisions (24). To date, these DCE studies investigating preventative dental care have been conducted in high-income countries, and no results are available in China. Considering China’s unique healthcare system, characterized by hierarchical service delivery and the coexistence of public and private dental providers, understanding public preferences for preventative dental care is particularly important. Therefore, this study employed a DCE to elicit the preferences for PDC among the Chinese population. Specifically, the study aimed to: (1) quantify the relative importance of different service attributes; (2) estimate willingness to pay for improvements in key service attributes; and (3) identify preference heterogeneity across population subgroups.

## 2 Materials and methods

### 2.1 Design

A DCE survey was developed following the good research practices for stated-preference studies as outlined by a report of the ISPOR Conjoint Analysis Good Research Practices Task Force (26). DCE assumes that products, services, or types of healthcare provision can be valued in terms of their constituent attributes (otherwise known as characteristics) and that respondents make choices in a utility-maximizing manner, preferring the alternative with the greatest utility to each individual. The method requires respondents to indicate a specific preference based on attribute levels in hypothetical scenarios. DCE has been used to elicit preferences for attributes of a specific product and could be a useful tool for investigating public preferences for PDC. A DCE design consists of four key stages: (1) identifying relevant attributes and associated levels, (2) designing the experiment and developing a questionnaire, (3) conducting the experiment and collecting data, and (4) analyzing the data. This study was approved by the Ethics Committee of the Affiliated Stomatology Hospital of Guangzhou Medical University (Approval No. LCYJ2024004). Informed consent was obtained from all participants.

### 2.2 Identification attributes and levels

Attributes and levels were identified through a three-step process, including targeted literature review, individual interviews, and expert consultations. An extensive literature search was conducted to identify the initial attributes and levels, among which the most commonly used attributes included waiting time, cost, personalized advice, preventative service time, and preventative effectiveness. To ensure the clinical relevance and meaningfulness of attributes, semi-structured interviews were conducted with 18 participants, including eight practicing dentists, six nurses, and four patients recruited from three stomatology hospitals through convenience sampling. Each interview lasted between 25 and 40 min (mean: 31 min), was conducted in a private meeting room, and was audio-recorded. Content analysis was performed to analyze the interview data, identifying themes and sub-themes. Detailed methodology of the literature review and interviews is provided in the Supplementary Appendix 1.

An initial list of attributes was generated through the above steps. The list delineated the characteristics associated with institutions, outcomes, and other relevant factors related to PDC. Second, three stomatology specialists were invited to evaluate the list of possible attributes. All attributes were required to be measurable, actionable, and realistic for policy implementation, while avoiding subjective or personalized characteristics. This procedure yielded ten attributes, including service effectiveness, risk of adverse reactions, attitude of provider, care provider, cost, hospital type, ability to get an appointment, waiting environment, waiting time, and travel time. Although the literature remains inconclusive regarding the number of attributes that should be included in a DCE, a systematic review indicates that most studies include 4–6 attributes (27). Therefore, there were six attributes in this study. Subsequently, 15 experts, including three public health specialists, seven oral nursing experts, and five stomatology professionals, independently ranked the importance of each candidate attribute using a five-point scale and provided reasons for excluding attributes. The rating results are presented in Supplementary Appendix Table 4. Attributes ranked among the top six by at least 60% of experts were retained for inclusion in the DCE (28). Four of the ten candidate attributes were excluded from the final design. “Travel time” was excluded because China has established a “15-min healthcare service circle,” meaning that residents can access nearby healthcare services within 15 min. “Waiting time” and “ability to get an appointment” varied by hospital class and were excluded. “Waiting environment” was excluded because of its low ranking. Six attributes most relevant to PDC were selected: (1) hospital type, (2) care provider, (3) attitude of provider, (4) service effectiveness, (5) risk of adverse reactions, and (6) cost. Hospital type, care provider, and attitude of provider represent service delivery factors. PDC quality is measured by service effectiveness and risk of adverse reactions. Cost is the monetary attribute that can be used to estimate willingness-to-pay for other attributes. The levels for all attributes were set to be consistent with previous studies. Detailed definitions of the attributes and levels are presented in Table 1.

**Table 1:** Detailed definitions of attributes and levels.

Attribute	Definition	Levels	Explanation
Hospital type	The types of dental service institutions.	Small private clinics	A privately-run dental clinic.
		High-level private providers	National private chain stomatology hospital.
		Community hospitals	Primary care institution.
		Class A tertiary hospitals	General hospital stomatology department and stomatology specialist hospital.
Care provider	According to the top-down order, the professional titles of Chinese doctors are from low to high.	physician	Junior professional title.
		Attending physician	Intermediate professional title.
		Chief physician	Senior professional title.
Attitude of provider	The service attitude of service providers during the diagnosis and treatment process.	poor	Inattentive.
		Normal	Normal.
		good	Warm and friendly.
Service effectiveness	The service effects that can be achieved through preventative oral services.	Poor	Failure to improve and alleviate oral health status.
		Fair	Partial improvement and relief oral health status.
		Good	Most improvement and relief oral health status.
		Very good	Significant improvement and relief oral health status.
risk of adverse reactions	The degree of adverse reactions (bleeding gums, tooth sensitivity, etc.) caused by preventative oral services to patients.	Severe	Fairly often occurring bleeding gums, tooth sensitivity, etc.
		Moderate	Occasionally occurring bleeding gums, tooth sensitivity, etc.
		Mild	Hardly ever occurring bleeding gums, tooth sensitivity, etc.
		None	Never occurring bleeding gums, tooth sensitivity, etc.
Cost	PDC cost per patient per year.	RMB 200	The annual cost of PDC is RMB 200.
		RMB 400	The annual cost of PDC is RMB 400.
		RMB 600	The annual cost of PDC is RMB 600.

### 2.3 Experimental design and questionnaire development

A full factorial design generated 1,728 (3^3^ × 4^3^) hypothetical scenarios and 1,492,128 [=(1728 × 1727)/2] pairwise choice tasks. To maximize the efficiency and precision of the design, Stata 17.0 was used to generate a D-efficient design limiting the number of choice sets to 24, which were randomly divided into 2 blocks, each containing 12 choice sets. Each choice set included two hypothetical service options and an opt-out alternative (“neither”). Respondents were asked to consider 2 PDC scenarios as realistic alternatives in each choice set and to choose the PDC scenario that appealed most to them. Subsequently, respondents were asked to choose between their preferred PDC protocol and no PDC. This “opt out” question is necessary because the PDC is a preventative intervention and, as in real life, the respondents are not obliged to undertake PDC. The ‘neither’ option prevented a forced choice set, thereby reducing the overestimation of attribute influence (29). We conducted an internal consistency test to ensure the data robustness. Each questionnaire version contained a dominant choice set, in which all the attribute levels of service A were superior to those of service B. Respondents were considered to have passed the consistency test if they chose service A.

**Figure 1:**
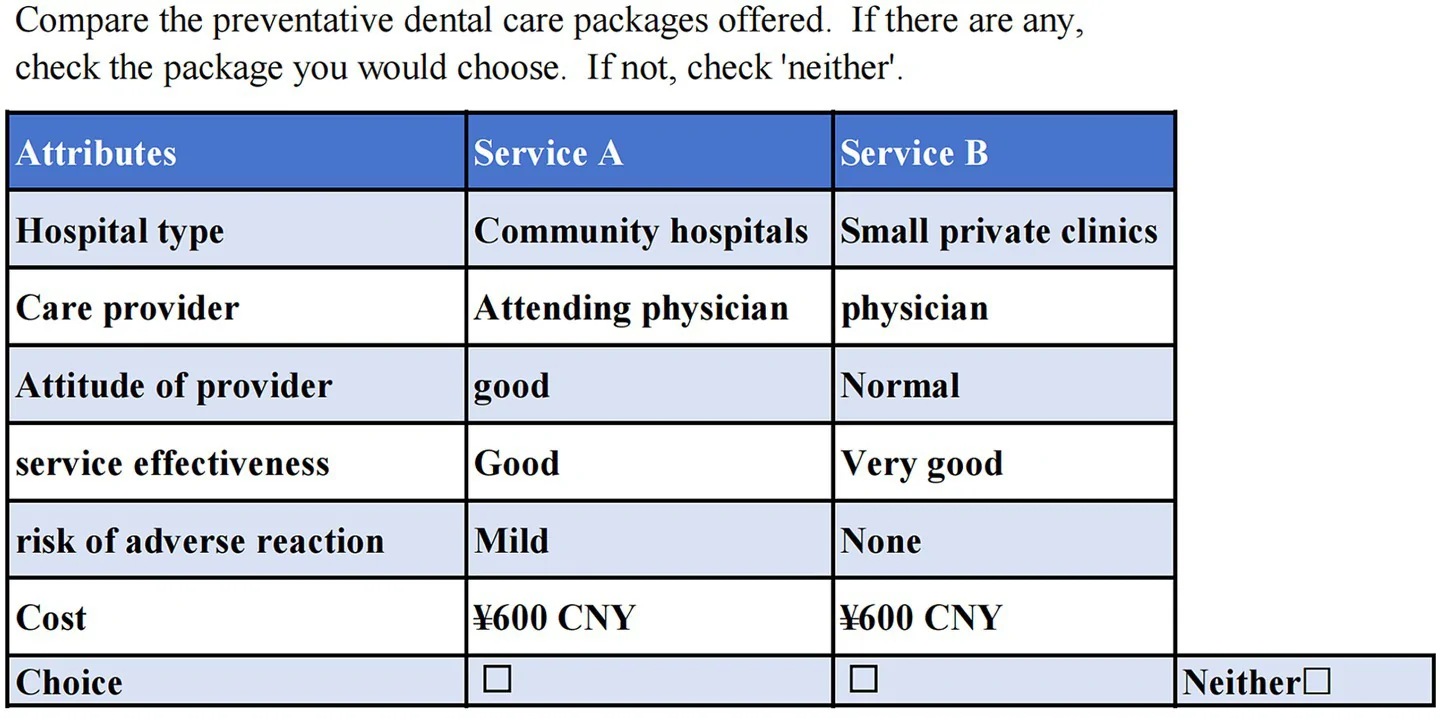
Example choice task from the discrete-choice experiment.

The survey comprised two sections. Section 1 included demographic questions (age, sex, household registration, education, marital status, etc.), as well as questions on their experience using dental care services and current oral health status. Section 2 described the choice tasks, explained the attributes and levels, clarified the implications of selecting the opt-out alternative, provided a completed example, and asked respondents to complete 13 choice tasks (including a dominant choice). The inclusion criteria required respondents who are over 18 years old, understand Chinese, and independently complete the questionnaire. To enhance the clarity, comprehensibility, and feasibility of the questionnaire, 20 respondents were recruited to complete the pilot test. The respondents required an average of 9.6 min to complete the questionnaire. We made minor changes to the layout, and the questionnaire was considered appropriate in length and easily understood by respondents through the pilot study. Data from the pilot tests were excluded from the final analysis. Figure 1 illustrates an example of a choice task.

### 2.4 Data collection

This survey was conducted at three stomatology hospitals in South China from February to October 2025. Participants were recruited using convenience sampling. Trained researchers carried out the face-to-face interviews. Only participants aged 18–60 years were included in the questionnaire, as this group has the independence and integrity to make reasonable comparative choices. Adults over 60 years were excluded, as they have more severe oral problems and preventative oral services may not be a priority for them.

The sample size was calculated using the rule of thumb formula proposed by Johnson and Orme. N > 500c/(t × a), where N is the sample size; c is the largest number of levels for any attribute (c = 4), t is the number of choice tasks (*t* = 12), and a is the number of alternatives (a = 2) (30). Despite the minimum required sample size of 83 yielded by this formula, we augmented the size in anticipation of subgroup analysis necessitated by preference heterogeneity. Accounting for the two questionnaire versions, we set the target sample size at 250. To ensure data quality, strict quality control standards were applied to filter the data. Questionnaires were excluded if respondents met any of the following exclusion criteria: (1) maintained uniform options from beginning to finish (10 items were deleted); (2) failed the consistency test (12 items were deleted); (3) submitted an incomplete questionnaire (7 items were deleted); (4) completed the questionnaire within less than 9.6 min (15 items were deleted). Any questionnaire meeting any of the above criteria was excluded from the final data analysis.

### 2.5 Statistical analysis

Descriptive statistics for demographic variables were calculated using SPSS version 27.0. Conditional logit and mixed logit models were performed using Stata version 17.0. Each choice set was treated as an observation. The attributes served as independent variables, while respondents’ choices (option A, option B, or option ‘neither’) served as the dependent variable. Mixed logit models were employed to analyze the DCE data, accounting for preference heterogeneity across the sample by treating coefficients as random effects. Based on random utility theory, the mixed logit model assumes that a respondent’s utility for a choice is determined by a combination of the fixed utility of observable attributes, random utility, error terms from unobserved attributes, and differences in individual preferences. The nonlinear relationship test was conducted with cost as a categorical variable, and the results showed a linear relationship. Therefore, cost was treated as a continuous variable, while other attributes were considered categorical variables. Dummy coding was used to code the attribute levels. The alternative-specific constant (ASC) was used to capture the utility of the “neither” option. Statistical significance was set at *p* < 0.05.

The utility U of respondent i in choice task t related to the choice of alternative j can be expressed as follows: U_ijt_ = V_ijt_ (X_ijt_, *β*_i_) + ε_ijt_, where V_ijt_ is the fixed term of Uijt, X_ijt_ is a vector of alternative-specific attribute levels, β_i_ represents the coefficient, and ε_ijt_ is the error term. A positive (negative) β coefficient indicates that the attribute-level combination is preferred (not preferred) relative to the reference level. Moreover, larger absolute β coefficients indicate stronger relative preference for a given attribute level compared with the reference category. Mean relative utility (β) and standard deviation (SD) were considered significant if their 95% confidence intervals (95% CI) excluded the null. To ensure data quality, samples that passed the consistency test were selected. Conditional logit models were also estimated for the robustness analysis. The model fits were evaluated based on the Akaike information criterion (AIC) and Bayesian information criterion (BIC) statistics to identify optimal models. Relative importance scores (RIS) were calculated to quantify the importance weight of each attribute, calculated by dividing the difference in utility between the lowest and highest levels of that attribute by the sum of all attribute differences. Additionally, willingness to pay (WTP) for attribute levels was calculated based on the cost of continuous attributes. WTP is an economic concept that measures the optimal price that an individual is willing to pay for a product. These WTP values provide a more easily comprehensible insight into the relative significance respondents attached to the pertinent DCE attributes in comparison to the *β* coefficients. Latent class analysis (LCA) was subsequently performed to identify the preference heterogeneity across respondent subgroups (31). Using the expectation–maximization algorithm, model fit was evaluated using the Akaike information criterion (AIC) and the Bayesian information criterion (BIC), with lower AIC and BIC values indicating better model fit.

## 3 Results

### 3.1 Demographic characteristics

A total of 277 respondents were included in the final analysis. Among them, 148 and 129 respondents completed questionnaire versions 1 and 2, respectively. The mean age of the participants was 40.0 years, and 66.4% were female. Approximately 61.4% of the respondents had urban household registration. Overall, 37.6% had a university degree. Most respondents (93.1%) were covered by basic medical insurance, and 35.3% reported a monthly per capita household income of RMB 10,000 and above. Regarding oral healthcare utilization, 39.7% of respondents had visited a dentist within the previous 6 months, and 45.1% rated their oral health status as fair. The sociodemographic characteristics of the participants are summarized in Table 2.

**Table 2:** Demographic characteristics of participants (*n* = 277).

Characteristic	Item	No (%)
Sex	Male	93(33.6%)
	Female	184(66.4%)
Age, y, mean (±SD)		40.0(±13.0)
Household registration	Urban	170(61.4%)
	Rural	107(38.6%)
Educational attainment	Junior middle school or below	48(17.3%)
	High school	38(13.7%)
	Vocational Diploma	87(31.4%)
	University	104(37.6%)
Marital status	Unmarried	84(30.3%)
	Married	180(65.0%)
	Divorce	11(4.0%)
	Widowed	2(0.7%)
Employment	Student	39(14.1%)
	Any paid employment	168(60.7%)
	Freelancer	25(9.0%)
	Retirement	36(13.0%)
	Unemployed or seeking work	9(3.2%)
Per capita monthly household income	RMB 2999 and below	32(11.6%)
	RMB 3000–4,999	72(26.0%)
	RMB 5000–9,999	75(27.1%)
	RMB 10000 and above	98(35.3%)
Medical insurance	No medical insurance	19(6.9%)
	Employee medical insurance	173(62.4%)
	Resident medical insurance	85(30.7%)
The time of the last dentist visit	3 months	58(21.0%)
	6 months	110(39.7%)
	12 months	56(20.2%)
	24 months	53(19.1%)
Self-reported oral health state	Very good	18(6.5%)
	Good	81(29.2%)
	Fair	125(45.1%)
	Poor	42(15.2%)
	Very poor	11(4.0%)

### 3.2 Preferences of the general population for PDC

A total of 9,972 observations were included in the preference analysis (277 participants × 12 completed choice tasks × 3 choice task alternatives). Both conditional logit and mixed logit models demonstrated statistically significant coefficients for all attribute levels relative to reference level, with the exception of the “chief physician” in the conditional logit model (shown in Tables 3, 4). These findings indicated that all attributes significantly influenced respondents’ choices. The mixed logit model demonstrated better model fit based on lower AIC (5678.117 < 6137.442) and BIC values (5879.928 < 6245.555) (see Table 5). The RIS for each attribute was calculated based on the estimated parameters of the mixed logit model. According to relative preference strength, the six attributes were ranked as follows: hospital type (34.16%), risk of adverse reactions (25.83%), service effectiveness (17.61%), cost (10.88%), attitude of provider (6.84%), and care provider (4.68%).

**Table 3:** Conditional logit model of preferences for PDC among general population.

Attributes	*β*	*SE*	*P*	*95%CI*
ASC	−1.179	0.168	<0.001	−1.508--0.850
Cost	−0.001	0.000	<0.001	−0.002−−0.001
Hospital type				
Small private clinics	Ref.			
High-level private providers	0.408	0.084	<0.001	0.243–0.574
Community hospitals	0.872	0.092	<0.001	0.692–1.052
Class A tertiary hospitals	1.581	0.094	<0.001	1.397–1.765
Care provider				
Physician	Ref.			
Attending physician	0.115	0.056	0.039	0.006–0.224
Chief physician	0.144	0.074	0.052	–0.001 – 0.289
Attitude of provider				
Poor	Ref.			
Normal	0.332	0.070	<0.001	0.195–0.468
Good	0.294	0.070	<0.001	0.158–0.431
Service effectiveness				
Poor	Ref.			
Fair	0.623	0.091	<0.001	0.445–0.800
Good	0.679	0.092	<0.001	0.498–0.859
Very good	0.982	0.096	<0.001	0.794–1.170
Risk of adverse reactions				
Severe	Ref.			
Moderate	0.635	0.093	<0.001	0.452–0.817
Mild	1.053	0.096	<0.001	0.864–1.242
None	1.333	0.098	<0.001	1.141–1.524
Nob = 9,972				
AIC = 6137.442				
BIC = 6245.555				

ASC (Opt-out), a specific constant item for opt-out; β, the average preferences of the study population; SE, standard error; ASC, alternative specific constant; Ref, reference level; CI, Confidence Interval; Nob, Number of observations; AIC, Akaike information criterion; BIC, Bayesian information criterion.

**Table 4:** Mix logit model of preferences for PDC among general population.

**Attributes**	***β***	***SE***	***P***	***95%CI***	***SD***
ASC	−1.785	0.224	<0.001	−2.224--1.345	
Cost	−0.002	0.000	<0.001	−0.002--0.001	
Hospital type					
Small private clinics	Ref.				
High-level private providers	0.525	0.132	<0.001	0.267–0.783	0.111; p < 0.001
Community hospitals	1.231	0.143	<0.001	0.952–1.511	0.144; P < 0.001
Class A tertiary hospitals	2.512	0.189	<0.001	2.142–2.882	0.159; P < 0.001
Care provider					
Physician	Ref.				
Attending physician	0.269	0.092	0.004	0.089–0.450	0.096; p < 0.001
Chief physician	0.344	0.110	0.002	0.128–0.560	0.245; *p* = 0.280
Attitude of provider					
Poor	Ref.				
Normal	0.503	0.101	<0.001	0.305–0.701	0.128; *p* = 0.005
Good	0.413	0.096	<0.001	0.225–0.600	0.144; *p* = 0.688
Service effectiveness					
Poor	Ref.				
Fair	0.744	0.130	<0.001	0.488–1.000	0.133; P < 0.001
Good	0.795	0.136	<0.001	0.529–1.061	0.127; *p* < 0.001
Very good	1.295	0.136	<0.001	1.029–1.560	0.202; *p* = 0.221
Risk of adverse reactions					
Severe	Ref.				
Moderate	0.572	0.142	<0.001	0.293–0.851	0.126; P < 0.001
Mild	1.512	0.149	<0.001	1.219–1.804	0.133; P < 0.001
None	1.899	0.152	<0.001	1.600–2.198	0.141; P < 0.001
Nob = 9,972					
Log likelihood = −2811.058					
AIC = 5678.117					
BIC = 5879.928					

**Table 5:** Goodness-of-fit test results.

Model	Obs	ll(null)	ll(model)	df	AIC	BIC
Conditional logit	9,972	−3651.787	−3053.721	15	6137.442	6245.555
Mix logit	9,972	−3053.721	−2811.058	28	5678.117	5879.928

The data are available from the corresponding author on reasonable request.

**Figure 2:**
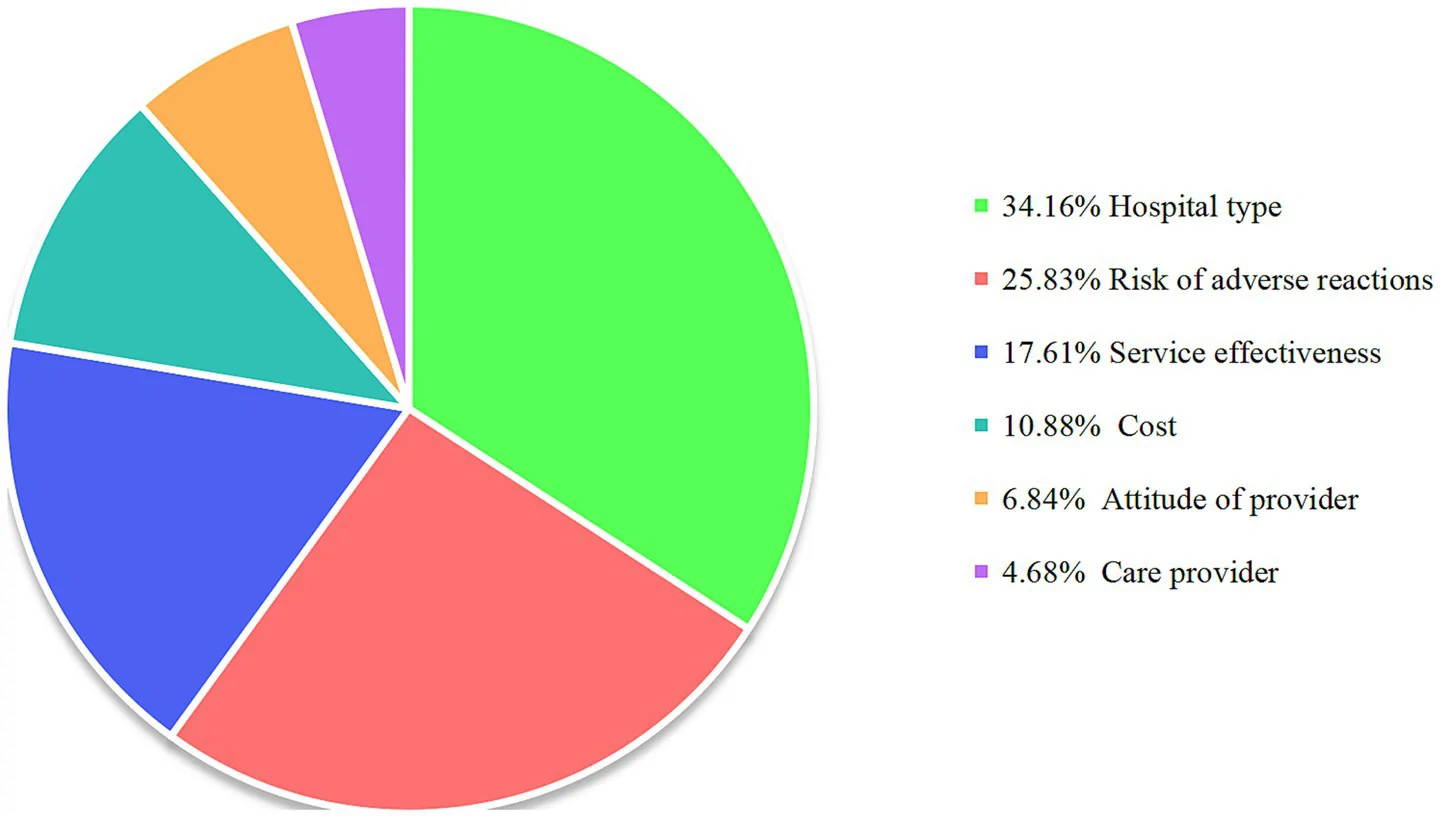
The relative importance scores of each attribute (%).

**Figure 3:**
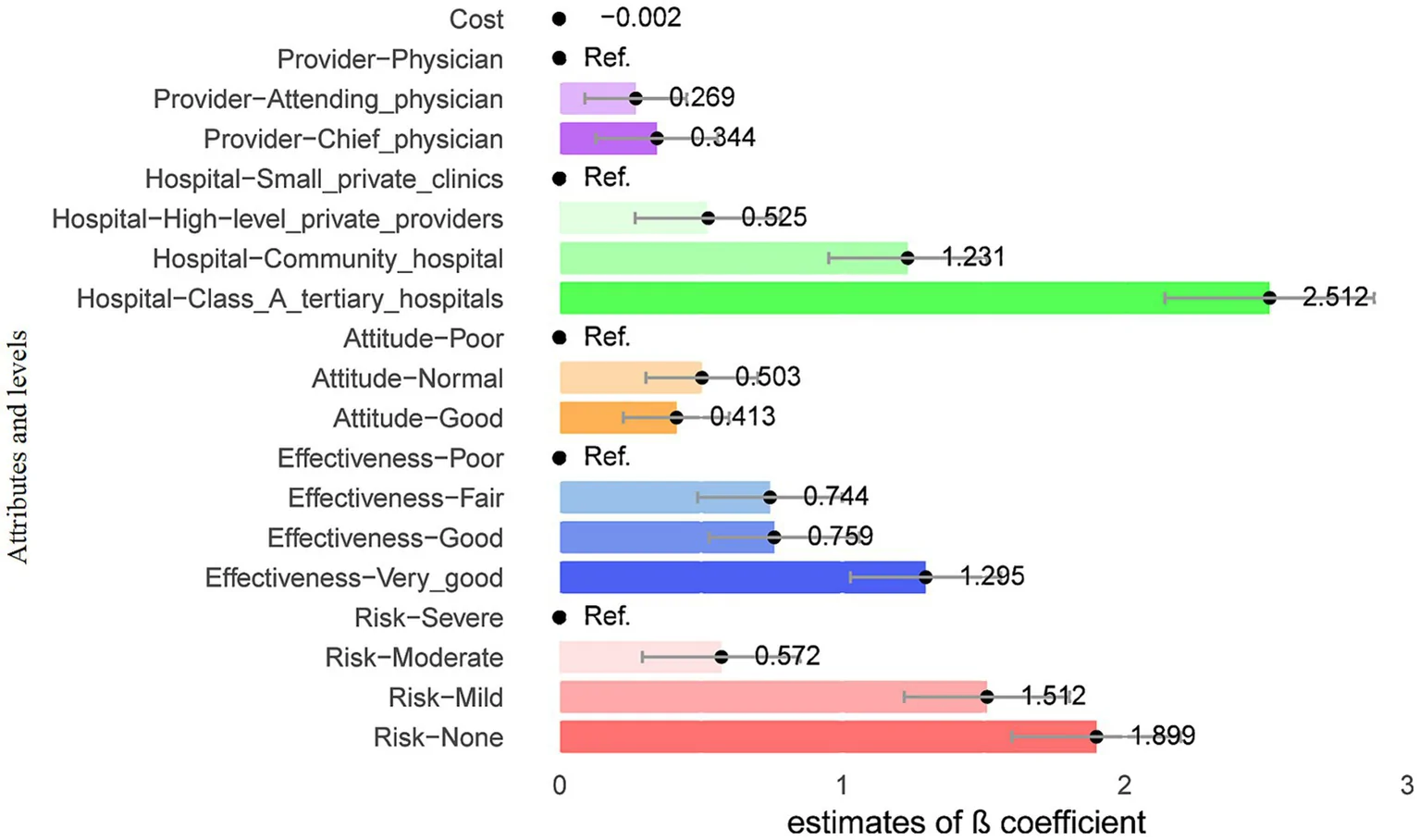
Preference of the general population for PDC.

Significant SDs were observed for all attribute levels, indicating substantial preference heterogeneity among respondents. Hospital type emerged as the most influential attribute, accounting for 34.16% of the importance weight. Compared with small private clinics, respondents showed the strongest preference for class A tertiary hospitals (*β* = 2.512), followed by community hospitals (*β* = 1.231). The second most critical factor was risk of adverse reactions (RIS = 25.83%). Regarding the risk of adverse reactions, respondents strongly preferred services associated with no risk (*β* = 1.899), although services with a mild risk remained acceptable (*β* = 1.512). Service effectiveness also substantially influenced respondents’ preferences (*β* = 1.295). Respondents preferred PDC with lower risks of adverse reactions and greater service effectiveness. The attitude of provider (*β* = 0.503) and care provider (*β* = 0.344) exerted comparatively smaller effects on respondents’ preferences. Cost was negatively associated with respondents’ preferences (*β* = −0.002), indicating that higher costs reduced the likelihood of choosing PDC. The estimated coefficients and RIS values for each attribute are presented in Figures 2, 3 and Table 4.

### 3.3 Estimates of WTP

The WTP estimates for each attribute level are presented in Table 6. The WTP estimates indicated that hospital type was the most highly valued attribute among respondents’ preferences for PDC. For hospital type, respondents were willing to pay an additional RMB 330.45 to upgrade from small private clinics to high-level private providers, RMB 775.22 to upgrade to community hospitals, and RMB 1581.90 to upgrade to class A tertiary hospitals. If the risk of adverse reactions was upgraded from severe to none, respondents were willing to pay an additional RMB 1195.8. When service effectiveness improved from poor to very good, respondents were willing to pay an additional RMB 815.12. A similar trend was observed for care provider. Notably, WTP for attitude of provider decreased as the attitude improved, indicating that respondents were less willing to pay additional cost or further improvements in provider attitude.

**Table 6:** Willingness to pay for PDC (*n* = 277).

Attributes		WTP (RMB)		
		WTP	95%CI	
Hospital type	Small private clinics→ High-level private providers	330.45	132.38	528.52
	Small private clinics→ Community hospitals	775.22	456.67	1093.77
	Small private clinics→ Class A tertiary hospitals	1581.90	1001.50	2162.30
Care provider	Physician→ Attending physician	169.67	39.92	302.41
	Physician→ Chief physician	216.45	58.64	374.25
Attitude of provider	Poor→ Normal	316.61	153.15	480.07
	Poor→ Good	259.78	121.14	398.42
Service effectiveness	Poor→ Fair	468.52	260.65	676.39
	Poor→ Good	500.58	281.46	719.70
	Poor→ Very good	815.12	517.01	1113.23
Risk of adverse reactions	Severe→ Moderate	360.21	185.63	534.81
	Severe→ Mild	951.85	657.15	1246.56
	Severe→ None	1195.89	820.00	1571.76

### 3.4 Latent class analysis model

Table 7 presents the results of the fitting indices of the latent class models (two to five classes). Both the AIC and BIC decreased as the number of latent classes increased. Considering both the model simplicity and interpretability, the three-class model was chosen as the optimal model. The results of the three-class model are presented in Table 8. The upper section reports the regression coefficients for each attribute within each latent class, whereas the lower section summarizes the association between latent class membership and respondents’ sociodemographic characteristics. Each profile was labeled based on comparisons of attribute probabilities, as follows:

**Table 7:** Model fit statistics for latent class models.

**Number of latent classes**	2	3	4	5
Log-likelihood function	−2694.59	−2514.02	−2429.12	−2379.61
AIC	5451.19	5122.05	4984.23	4917.22
BIC	5563.53	5292.38	5212.55	5203.52
Size of the smallest group (proportion of sample)	45.14%	18.21%	12.21%	4.70%

AIC: Akaike information criterion; BIC: Bayesian information criterion.

**Table 8:** Results of parameter estimation of LCA.

Preference	Class 1 (probability 39.0%)		Class 2 (probability 17.8%)		Class 3 (probability 43.2%)	
	*β*	SE	*β*	SE	*β*	SE
ASC	**−6.145** ^***^	0.475	**−3.277** ^**^	0.944	**3.598** ^***^	0.492
Cost	0.000	0.000	**−0.005** ^***^	0.001	**−0.002** ^***^	0.000
Hospital type						
Small private clinics	Ref.		Ref.		Ref.	
High-level private providers	0.049	0.177	**3.201** ^***^	0.809	**0.373** ^**^	0.113
Community hospitals	**0.714** ^***^	0.185	**4.581** ^***^	0.826	**0.671** ^***^	0.139
Class A tertiary hospitals	**0.970** ^***^	0.190	**6.617** ^***^	0.880	**1.553** ^***^	0.157
Care provider						
Physician	Ref.		Ref.		Ref.	
Attending physician	0.138	0.116	**0.509** ^*^	0.237	0.012	0.082
Chief physician	**0.535** ^**^	0.157	**0.894** ^**^	0.315	**0.226** ^*^	0.115
Attitude of provider						
Poor	Ref.		Ref.		Ref.	
Normal	**1.107** ^***^	0.155	**0.687** ^*^	0.297	0.118	0.098
Good	**1.043** ^***^	0.165	**0.894** ^**^	0.286	0.158	0.106
Service effectiveness						
Poor	Ref.		Ref.		Ref.	
Fair	**1.824** ^***^	0.248	0.648	0.333	**0.356** ^**^	0.112
Good	**2.198** ^***^	0.244	−0.116	0.366	**0.490** ^***^	0.122
Very good	**2.687** ^***^	0.240	−0.165	0.431	**0.685** ^***^	0.140
Risk of adverse reactions						
Severe	Ref.		Ref.		Ref.	
Moderate	**2.146** ^***^	0.320	−0.427	0.416	**0.454** ^***^	0.125
Mild	**3.442** ^***^	0.321	−0.411	0.384	**0.579** ^***^	0.136
None	**4.125** ^***^	0.333	−0.017	0.372	**0.570** ^***^	0.136
Influence factors						
Age	−0.025	0.018	0.029	0.024		
Sex ^a^	−0.055	0.334	−0.091	0.435		
Educational attainment ^b^	0.589	0.445	−0.944	0.596		
Marital status ^c^	−0.528	0.439	−0.104	0.706		
Income ^d^	**−1.107** ^*^	0.431	0.690	0.580		
Medical insurance ^e^	−0.103	0.583	1.015	1.154		
Oral disease ^f^	0.133	0.402	−0.446	0.557		
Household registration ^g^	0.032	0.330	0.882	0.494		
Oral health state ^h^	−0.649	0.430	0.808	0.495		

β, the average preferences of the study population; SE, standard error; ASC, alternative specific constant; Ref, reference level; ^***^p < 0.001, ^**^p < 0.01, ^*^p < 0.05. In this section, only the significant values are highlighted in bold. The cost is a continuous variable. a, compared with males; b, compared with shorter length of education (high school and below); c, compared with unmarried; d, compared with per capita monthly household income less than 5,000 RMB; e, compared without medical insurance; f, compared without oral disease; g, compared with urban household registration; h, compared with self-reported oral health state “good”.

The respondents (39.0%) in class 1 prioritized service effectiveness and risk of adverse reactions. They placed significant emphasis on the service effectiveness of PDC, while simultaneously showing an emphasis on treatment quality and safety, so this group was labelled the “quality-oriented” class.

The respondents (17.8%) in class 2 showed the strongest preference for hospital type, particularly class A tertiary hospitals. This group was therefore labelled the “institution-oriented” class.

The respondents (43.2%) in class 3 demonstrated positive preferences for hospital type, service effectiveness, and risk of adverse reactions, with relatively balanced importance across these three attributes. Notably, the alternative specific constant (ASC) of this class had a positive coefficient, indicating that the general population preferred to have PDC over none. This group was therefore classified as the “comprehensive consideration” class.

Among all sociodemographic variables included in the model, only household income was significantly associated with latent class membership. Respondents with a monthly per capita household income exceeding RMB 5000 were more likely to belong to the “quality-oriented” class.

## 4 Discussion

To our knowledge, this is the first discrete choice experiment to investigate public preferences for preventative dental care in China. Overall, hospital type was the most influential attribute affecting respondents’ PDC preferences, followed by risk of adverse reactions and service effectiveness. WTP analysis further demonstrated that respondents were willing to pay more for PDC provided by class A tertiary hospitals. In addition, LCA identified three distinct preference classes, highlighting substantial heterogeneity in public preferences regarding PDC.

Hospital type emerged as the most influential attribute in respondents’ decision-making regarding PDC selection. Although private dental institutions account for approximately 80% of dental care facilities in China and community health centers provide geographically accessible services through the “15-min healthcare service circle,” public hospitals remain dominant (28, 32). Several factors may explain this finding. First, public tertiary hospitals are generally perceived as providing higher-quality healthcare because of their greater concentration of experienced clinicians, advanced diagnostic equipment, and well-established referral systems (32–34). Second, hospital level may serve as a proxy for perceived healthcare quality. Patients often associate larger public hospitals with greater clinical competence and more reliable treatment outcomes. Third, higher medical insurance reimbursement rates may encourage patients to seek care in tertiary hospitals. Previous experience with public hospitals may also reinforce subsequent healthcare-seeking behaviors through familiarity and habit formation (35). Nevertheless, Zhu et al. reported a preference for high-level private hospitals among participants (32). This discrepancy may be attributable to differences in study populations, as most respondents in that study had previous experience with private healthcare providers. One important implication is that changing traditional public perceptions may be challenging. Further efforts to modify public perceptions and behaviors regarding public-private healthcare provision might need to combine policy incentives with effective education programs that explain all aspects of oral services provided by private providers.

Risk of adverse reactions and service effectiveness were the second and third most influential attributes, respectively. Respondents prioritized risk minimization over efficacy maximization. This finding is consistent with previous literature suggesting that individuals may exhibit loss avoidance preference in uncertain environments (29, 36). Severe adverse reactions significantly reduce the acceptance rates of services. Service effectiveness, closely tied to medical technology levels, is also important (33). Therefore, decision-makers should consider both the potential health and non-health benefits of services when allocating resources. Cost, attitude of provider, and care provider played relatively minor roles in PDC selection (29). First, “patients’ choices of healthcare providers are not always rational and straightforward” (37). These decisions are determined by a complex interplay between patient and provider characteristics. There may also be an exposure effect that further compounds this complexity; that is, people tend to develop a preference simply because of familiarity (38). Particularly within the guanxi-oriented (relationship-oriented) society context in China, established patient-provider relationships may be more influential than physicians’ qualifications (39). Cost exhibited relatively low importance (29). Increasing household income and improvements in oral health literacy may have reduced respondents’ sensitivity to treatment costs (40). Second, because DCEs are based on hypothetical scenarios, respondents may place greater emphasis on desirable service attributes than on monetary considerations.

The WTP analysis translated respondents’ preferences into monetary values, providing information that may inform future pricing and payment mechanisms (41). The high WTP associated with class A tertiary hospitals further reinforced the strong preference for high-level public healthcare institutions observed in the preference analysis. Respondents were willing to pay approximately RMB 1500 for PDC provided by class A tertiary hospitals. Choosing public hospitals might reflect a more general support for the public health system as a whole. The elevated WTP estimates observed in this study may stem from two factors. First, DCE relies on hypothetical scenarios, and payment levels are determined subjectively by patients (42, 43). Secondly Guangzhou is a large city with relatively higher income compared to the national average, which may also contribute to higher acceptable costs for PDC.

Latent class analysis identified three distinct preference patterns, demonstrating substantial heterogeneity in respondents’ decision-making. Although preference priorities differed across latent classes, all three groups consistently valued hospital type, suggesting a broadly shared preference for public tertiary healthcare institutions. This confirms the path-dependent phenomenon of China’s hierarchical medical system (44). Therefore, policymakers should consider the preference heterogeneity when designing preventative dental care programs and develop targeted interventions for different population groups.

## 5 Limitations

This study has several limitations. First, participants were recruited from three stomatology hospitals in Guangdong Province, which may limit the generalizability of the findings to other regions and healthcare settings in China. Guangdong Province is one of the most populous and economically developed regions in China and attracts a large migrant population, which may partially enhance the diversity of the study sample. Moreover, because participants were recruited from stomatology hospitals, individuals who rarely or never seek preventative dental care may have been underrepresented. Future studies should recruit participants from community settings and general hospitals to improve population representativeness. Second, DCEs only allow participants to make choices in hypothetical scenarios and may not fully capture actual decision-making behavior. Similarly, willingness-to-pay estimates reflected respondents’ stated preferences rather than their actual willingness to pay and may therefore be subject to hypothetical bias. Third, this study analyzed only the overall public preference and did not examine interaction effects between attributes and demographic subgroups. Subsequent studies could conduct more detailed analysis according to different demographic characteristics. Finally, although face-to-face interviews may increase the risk of social desirability bias, trained interviewers and privacy protocols may have mitigated this problem.

## 6 Conclusion

This study provides demand-side evidence regarding public preferences for preventative dental care among urban Chinese adults. The findings demonstrate that individuals’ choices regarding PDC are primarily influenced by hospital type, risk of adverse reactions, service effectiveness, cost, attitude of provider, and care provider, while also revealing heterogeneity in preferences across different groups of people. Understanding these factors can help public health authorities design targeted interventions to improve PDC acceptance. Future research should continue to explore these dynamics in different populations and contexts to further enhance understanding of PDC behavior.

## Data Availability

The original contributions presented in the study are included in the article/Supplementary material, further inquiries can be directed to the corresponding author/s.
