# Supplementary material for: Public preferences for preventative dental care: a discrete choice experiment in China
Source: Front Public Health. 2026 Aug 31;14:1886047. doi: 10.3389/fpubh.2026.1886047 (PMC13572415; doi:10.3389/fpubh.2026.1886047)
Supplement: Supplementary file 1 [file Data_Sheet_1.pdf]

Supplementary Appendix 1

Public Preferences for Preventative Dental Care: A discrete choice experiment

Contents

Table 1. Identification of Potential Attributes from the Targeted Systematic Review 2
Table 2. Results of Personal Interviews 6
Table 3. Potential Attribute Pool 8
Table 4. Result of Attribute Ratings by 15 Experts 9

A targeted literature search was conducted in June 2024 using the following databases: PubMed, Embase, Web of Science Core Collection, and China National Knowledge Infrastructure to identify studies related to patients' and public preferences for dental services. To obtain a recent overview of the literature, we searched for studies published during the previous 10 years (2014–2024). The search included (1) terms related to preventative dental care and (2) terms related to preferences and discrete choice experiments. The search terms were as follows: ("patient* preference*" OR "patient* priorit*" OR "public preference*" OR "discrete choice experiment" OR "DCE" OR "conjoint analysis" OR "stated preference*" OR "willingness to pay" OR "WTP") AND ("oral health*" OR "oral health care*" OR "dentistry*" OR "dental care*" OR "dental service*"). Additional literature was identified through a hand search in the reference lists of the included articles. Titles, abstracts and full texts of the identified studies were independently screened for relevance by two authors (BNC, ZCH) and ambiguous cases were discussed.

Table 1. Identification of Potential Attributes from the Targeted Systematic Review

No.	Potential attributes	Description	Relevant articles (Author, Year)
1	Painless	The dentist uses painless anesthesia.	Bech M(2011)
2	Waiting time	The time a patient has to wait from booking an appointment until being seen by the dentist.	Bahanan L(2023), Zhu J(2019), Sever I(2018), Kiiskinen U(2010), Sever I(2019)
3	Ability to obtain an appointment	The time required to obtain an appointment for dental services.	Bahanan L(2023)
4	Travel time	The time required for an individual to travel to the	Zhu J(2019), Kiiskinen U(2010), Wang L(2024), Bech M(2011)
5	Preventive service time	Service operating hours.	Bech M(2011), Wang L(2024)
6	Service cost	Cost of treatment (routine check-up plus cleaning).	Boyers D (2021), Zhu J (2019), Sever I (2018), Sever I (2019), Chebib N (2024), Wang L (2024), Kiiskinen U (2010), Bech M (2011), Vennedey V (2018)
7	Personalized advice	Detailed and personalized oral hygiene advice.	Sever I (2019), Boyers D (2021)<br>Sever I(2018)
8	Service provider	The professional title of the healthcare provider delivering the service, including physicians, attending physicians, and chief physicians.	Bahanan L (2023), Chebib N (2023), Sever I(2018), Sever I (2019)

Table 1 Continued

9	Frequency of visits	The frequency of visits for preventative dental services.	Kiiskinen U (2010), Vennedey V(2018), Boyers D (2021)
10	Hospital type	The type and level of healthcare institutions.	Bahanan L(2023), Zhu J(2019), Chebib N(2024), Sever I(2018), Sever I(2019)
11	Waiting environment	The environment of the dental facility waiting area.	Zhu J(2019)
12	Centre	Dental center.	Bech M(2011)
13	Dental staff attitudes	Dental staff attitudes toward patients.	Sever I(2018), Sever I(2019)
14	Explanation of dental treatment	Whether explanations of dental treatment are sufficiently detailed.	Sever I(2018), Sever I(2019), Boyers D(2021)
15	Preventive effectiveness	The probability of reduction in dental caries, the aesthetic effect, and the possibility of tooth loss over the next 10 years.	Boyers D (2021), Wang L (2024), Vennedey V (2018)
16	Choice of doctor	Patients can choose their preferred doctors.	Zhu J(2019)
17	Risk of adverse reactions	Complaints and symptoms (occasional gum bleeding," long teeth" due to gingival recession, and sensitive tooth neck).	Boyers D(2021), Vennedey V(2018)
18	X-ray	Whether Whether the dentist has access to a digital	Bech M(2011)
19	Distance	Distance to the dentist.	Bech M (2011)

Reference

Bahanan L, Bashkail F, Alghamdi A, Alhazmy A. Assessing Patients' Preferences for Preventive Dental Care: A Discrete Choice Experiment. Cureus. 2023 Aug 24;15(8):e44028. doi: 10.7759/cureus.44028. PMID: 37746415; PMCID: PMC10517430.

Bech M, Kjaer T, Lauridsen J. Does the number of choice sets matter? Results from a web survey applying a discrete choice experiment. Health Econ. 2011 Mar;20(3):273-86. doi: 10.1002/hec.1587. PMID: 20143304.

Boyers D, van der Pol M, Watson V, Lamont T, Goulao B, Ramsay C, Duncan A, Macpherson L, Clarkson J. The Value of Preventative Dental Care: A Discrete-Choice Experiment. J Dent Res. 2021 Jul;100(7):723-730. doi: 10.1177/0022034521989943. Epub 2021 Feb 4. PMID: 33541186; PMCID: PMC8217903.

Chebib N, Holmes E, Maniewicz S, Abou-Ayash S, Srinivasan M, McKenna G, Kossioni A, Schimmel M, Müller F, Brocklehurst P. Exploring preferences of older adults for dental services: A pilot multi-national discrete choice experiment. Gerodontology. 2024 Jun;41(2):220-230. doi: 10.1111/ger.12696. Epub 2023 Jun 13. PMID: 37309614.

Kiiskinen U, Suominen-Taipale AL, Cairns J. Think twice before you book? Modelling the choice of public vs private dentist in a choice experiment. Health Econ. 2010 Jun;19(6):670-82. doi: 10.1002/hec.1504. PMID: 19504546.

Sever I, Verbič M, Sever EK. Estimating willingness-to-pay for health care: A discrete choice experiment accounting for non-attendance to the cost attribute. J Eval Clin Pract. 2019 Oct;25(5):843-849. doi: 10.1111/jep.13095. Epub 2019 Jan 24. PMID: 30677196.

Sever I, Verbič M, Sever EK. Valuing the delivery of dental care: Heterogeneity in patients' preferences and willingness-to-pay for dental care attributes. J Dent. 2018 Feb;69:93-101. doi: 10.1016/j.jdent.2017.12.005. Epub 2017 Dec 11. PMID: 29242040.

Vennedey V, Derman SH, Hiligsmann M, Civello D, Schwalm A, Seidl A, Scheibler F, Stock S, Noack MJ, Danner M. Patients' preferences in periodontal disease treatment elicited alongside an IQWiG benefit assessment: a feasibility study. Patient Prefer Adherence. 2018 Nov 16;12:2437-2447. doi: 10.2147/PPA.S176067. PMID: 30510407; PMCID: PMC6248230.

Wang L, Chen Z, Chen S, Jin X, Hu L, Jiang J, Yu H, Xu J, Xu W. Optimisation of services to prevent dental caries for school-aged children in China: a discrete choice experiment. BMJ Open. 2024 Dec 23;14(12):e084776. doi: 10.1136/bmjopen-2024-084776. PMID: 39719292; PMCID: PMC11667323.

Zhu J, Li J, Zhang Z, Li H, Cai L. Exploring determinants of health provider choice and heterogeneity in preference among outpatients in Beijing: a labelled discrete choice experiment. BMJ Open. 2019 Apr 3;9(4):e023363. doi: 10.1136/bmjopen-2018-023363. PMID: 30948563; PMCID: PMC6500246.

Personal Interview Guide

Part 1: Interview Guide for the General Population

1. Have you experienced any oral health-related issues? If so, please specify them.
2. What do you think constitutes good oral health?
3. What does good oral health mean to you personally?
4. What measures do you think can be taken to maintain good oral health? (e.g., dental examinations, scaling and polishing, or personalized advice)
5. Have you ever visited a dental hospital for treatment or oral examination?
6. Please elaborate on your overall dental visit experience, including the pre-treatment, treatment and post-treatment stages.
7. Do you think it is necessary to visit a dental hospital regularly for preventative dental care? How often?
8. What motivates you to receive regular preventative dental care?
9. What prevents you from receiving regular preventative dental care?
10. What services do you think should be included in preventative dental care?
11. How much do you think each preventative dental care session should cost?
12. Do you have any suggestions regarding preventative dental care?

Part 2: Interview Guide for Medical Staff

1. How do you think dentists should help patients maintain good oral health?
2. Is the support currently provided by dentists sufficient to help patients maintain their oral health?
3. How often do patients need to receive preventative dental care?
4. What motivates patients to receive regular preventative dental care?
5. What prevents patients from receiving regular preventative dental care?
6. Do you have any suggestions for improving current preventative dental care services?

Table 2. Results of Personal Interviews

Theme	Sub-theme	Explanation	quotations
Service delivery	Service provider	Service providers include dental institutions and individual healthcarel practitioners. Dental institutions encompass public hospitals, private hospitals, and private clinics. Medical practitioners are generally categorized according to their professional titles, such as physicians, attending physicians, and chief physicians.	P1: "I would definitely choose specialized stomatological hospitals or public general hospitals as my first choice."<br>M2: "Generally, the main providers of scaling and polishing services are physicians, attending physicians, and chief physicians."<br>P2: "What matters most to me is the professionalism of the doctors."
	Treatment planning	Treatment planning involves scheduling intervals between visits and ensuring continuity of care. Most respondents recommended intervals of six months to one year between visits. Continuity of care refers primarily to having the same doctor oversee current and future preventive oral services. This continuity fosters trust and familiarity and enables doctors to better understand patients' oral health, allowing for more personalized treatment recommendations.	P3: "Regarding the interval between follow-up visits, six months to a year seems appropriate."<br>P6: "I prefer to have the same doctor responsible for my oral health. On one hand, I am familiar with and trust this doctor; on the other hand, they understand my oral condition better."
	Service cost	Service costs include financial and time costs. Financial costs refer to the monetary expense of preventive oral care, while time costs include travel time, waiting time, and treatment duration.	P1: "Considering my current financial situation, I am very concerned about the cost."<br>P2: "If the dental clinic is too far away, I am less likely to go."<br>P3: "I think when visiting the hospital, you have to wait a long time. There are many patients in public hospitals."<br>P4: "It was my first time having my teeth cleaned. I thought the process would be very thorough, but it only took about 10 minutes. Can my teeth really become clean in such a short time?"

Table 2 Continued

Expected and unexpected treatment outcomes	Oral health	The clinical benefits of preventive oral services include improved tooth cleanliness and relief from periodontal conditions.	P3: "After scale and polish, teeth become cleaner and more beautiful."<br>M3: "The tartar removal process can sometimes cause bleeding and a metallic taste or odor."
	Oral health-related quality of life	Improvements in oral health have a positive impact on mental well-being.	M4: "Good oral naturally contributes to better mental health."
	Adverse reactions	Adverse reactions to preventive oral care may include gum bleeding and tooth sensitivity.	P3: "The process of removing tartar may cause bleeding and a bloody odor."<br>M1: "Most patients are afraid of pain, so they hesitate to visit the dentist."

Table 3. Potential Attribute Pool

No.	Potential attributes	Description
1	Painless	The dentist uses painless anesthesia.
2	Waiting time	The waiting interval from appointment booking to clinical consultation.
3	Ability to obtain an appointment	The time required to obtain an appointment for dental services.
4	Travel time	The time required for an individual to travel to the dentist.
5	Service time	Service operating hours.
6	Cost	Cost of treatment (routine check-up plus cleaning).
7	Personalized advice	Detailed and personalized oral hygiene advice.
8	Service provider	The professional title of the health professional providing the service, including physicians, attending physicians, and chief physicians.
9	Frequency of visits	The frequency of visits for preventative dental services.
10	Hospital type	The type and level of healthcare institutions.
11	Waiting environment	The environment of the dental facility waiting area.
12	Centre	Dental center.
13	Dental staff attitudes	Dental staff attitudes toward patients.
14	Explanation of dental treatment	Whether explanations of dental treatment are sufficiently detailed.
15	Preventive effectiveness	The probability of reduction in dental caries, the aesthetic effect, and the possibility of tooth loss over the next 10 years.
16	Choice of doctor	Patients can choose their preferred doctors.
17	Risk of adverse reactions	Complaints and symptoms (occasional gum bleeding, "long teeth" due to gum , and sensitive tooth necks).
18	X-ray	Whether the dentist has access to a digital X-ray machine.
19	Distance	Distance to the dentist.
20	Continuity of care	The same doctor supervises current and future preventative dental services.
21	Oral health-related quality of life	Improvements in oral health have a positive impact on mental well-being.

Table 4. Result of Attribute Ratings by 15 Experts

Attribute	E1	E2	E3	E4	E5	E6	E7	E8	E9	E10	E11	E12	E13	E14	E15	Mean Score
Service effectiveness	5	4	5	5	4	5	3	4	4	4	5	4	5	5	5	4.467
Risk of adverse reactions	5	3	5	4	5	5	3	4	4	5	5	4	4	5	5	4.400
Attitude of provider	5	5	4	5	4	3	4	5	5	5	4	4	5	4	3	4.333
Care provider	4	4	3	4	5	3	3	5	4	4	5	5	4	5	5	4.200
Cost	3	4	5	4	3	3	3	5	5	4	5	5	4	5	4	4.133
Hospital type	4	4	3	3	4	4	4	4	3	4	5	3	3	5	4	3.800
Ability to obtain an appointment	3	4	3	4	4	3	3	4	3	5	5	4	4	3	3	3.667
Waiting time	4	4	5	3	4	2	3	3	2	4	4	4	4	5	3	3.600
Waiting environment	3	3	4	3	4	2	3	3	5	3	5	2	5	5	3	3.533
Travel time	3	3	4	4	3	2	3	2	4	3	4	4	4	1	3	3.133
